# Surrogate cytokine agonists: promising agents against COVID-19

**DOI:** 10.1038/s41392-022-01015-w

**Published:** 2022-05-06

**Authors:** Zemin Lin, Jianping Zuo, Shijun He

**Affiliations:** 1 Laboratory of Immunopharmacology, State Key Laboratory of Drug Research, Shanghai Institute of Materia Medica, Chinese Academy of Sciences, Shanghai, 201203 China; 2 University of Chinese Academy of Sciences, Beijing, 100049 China; 3 Laboratory of Immunology and Virology, Experiment Center for Science and Technology, Shanghai University of Traditional Chinese Medicine, 1200 Cailun Road, Shanghai, 201203 China

**Keywords:** Infection, Molecular medicine

Cytokines are small cell-signaling proteins which are critical coordinators for the immune response that takes place in infection and inflammation. A recent study published in Cell by Yen et al.^1^ demonstrated an innovative modular platform enabled engineering surrogate ligand for cytokine receptors therein displayed versatile functional activities. Further, the novel “cytokine med-chem” strategy they presented has been proved to be applicable to many cell surface transmembrane receptor systems, especially for the surrogate interferons (IFNs), that exhibited potent inhibition of the severe acute respiratory syndrome coronavirus 2 (SARS-CoV-2) with restrained pro-inflammatory effects. This research provided a powerful screening platform for cytokine drug discovery and particularly inspired the development of small molecule drugs for Corona virus disease-2019 (COVID-19).

COVID-19, caused by the SARS-CoV-2, is an acute viral infection, which has taken an enormous toll on human lives worldwide. One of the hallmarks of severe COVID-19 is low levels of antiviral type I and III IFNs and excessive production of pro-inflammatory and inflammatory cytokines^2^. It is therefore essential to enhance early protection through activating type I and III IFN systems and inhibit pro-inflammatory activities during the later stages of COVID-19^3^. However, a late administration of type I IFN aggravated disease in turn and increased mortality in some clinical trials, indicating a dual role of type I IFNs in host defense and immunopathology^4^. Furthermore, the shift of a particular cytokine from its pro-inflammatory to anti-inflammatory role is sensitive to factors such as the concentration, the presence of other cytokines, and the target cells. Thus, mitigating the cytokine pleiotropy through engineering small molecule agonists that act as surrogate ligands with drug-like properties could be a promising strategy for discovering novel molecules for COVID-19 therapy.

Nevertheless, cytokines transduce their signals through the type I single-pass transmembrane (TM1) receptors, which are distinct from the multi-pass transmembrane proteins like ion channels and G-protein-coupled receptors (GPCRs), thus great challenges aroused in small molecule agonist discovery because of the structural properties of TM1^5^. Until recently, Yen et al. presented a strategy to discover cytokine surrogate agonists by using modular ligands that exploit induced proximity and receptor dimer geometry as pharmacological metrics accommodating to high-throughput screening (HTS)^1^.

**Fig. 1:**
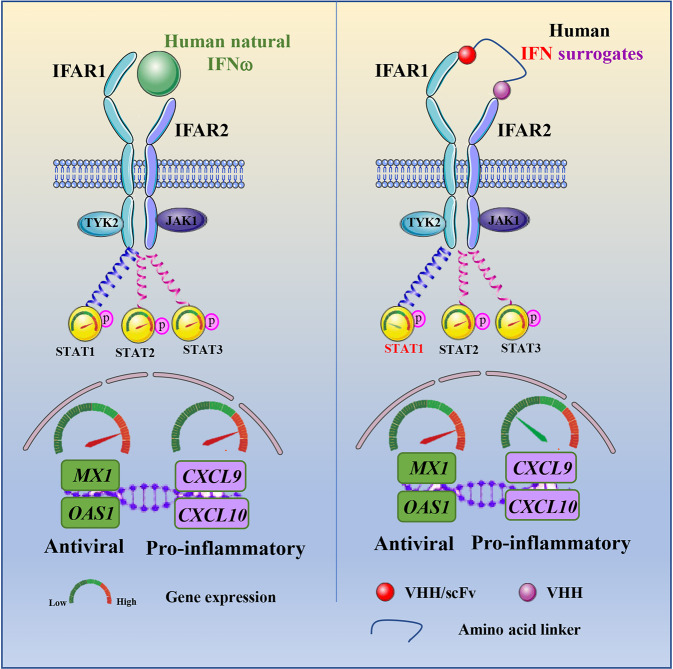
The bispecific type-I interferon surrogate ligands exhibit antiviral and pro-inflammatory effects discriminately. Human IFN surrogates ligands induced decreased activation of pSTAT1 but equivalent activation of pSTAT2 and pSTAT3 compared to the natural human IFNω in vitro. HIS agonists induced comparable levels of the antiviral genes as natural human IFNω but minimal expression of pro-inflammatory genes. VHH single variable domain of a heavy-chain, scFv single chain antibody fragment

Type I IFNs possess antiviral, immunomodulatory, and anti-proliferative activities involving 16 diverse sub-types that dimerize IFNAR1/IFNAR2 to activate certain signal transducer and activator of transcription (STATs). Yen and colleagues created surrogate agonists in the type I IFN system applying a collection of single variable domain of a heavy-chain (VHH) and single chain antibody fragment (scFv) binders to human IFNAR1 and IFNAR2, fused via 2 or 5 amino acid linkers (Fig. 1). They yielded 12 hits from a 60-member screening matrix through the sequential selection by surface plasmon resonance (SPR) analysis and activity assays based on STAT1 phosphorylation. A subset of the hits, termed as “human interferon surrogates” (HISs), induced decreased activation of pSTAT1 but comparable activation of pSTAT2 and pSTAT3 to the natural human IFNω (Fig. 1).

Antiviral assay was conducted in the study utilizing the recombinant SARS-CoV-2 engineered A549 lung epithelial cells and primary human airway epithelial cells to measure the virus replication. Yen and colleagues found that the antiviral potency of HIS agonists depended on the identity of the IFNAR1 binder, moreover, the HIS ligands preserved high levels of antiviral gene expression but elicited lower levels of pro-inflammatory gene expression in human primary airway epithelial cells (Fig. 1). Taken together, the surrogate IFN agonists generated by the approach employs the intrinsic signaling and functional plasticity of cytokines could be a potential therapeutic option for COVID-19 and other infection.

Altogether, Yen and colleagues proposed a “cytokine med-chem” approach rooted in principles of induced proximity, utilizing the structurally agonistic and modular platform, to develop cytokine surrogate agonists which may serve as novel starting points for investigating therapies of COVID-19 and many other diseases. In addition, the surrogate cytokine agonist platform allows tuning of cytokine signaling pathways and a tailored cellular response beyond simply recapitulating natural cytokine receptors thus will potentiate the full exploration for drug discovery.
